# IgG Idiotype Diversity Shapes Cytokine Profiles and Autoantibody Targets in HTLV-1 Clinical Outcomes

**DOI:** 10.3390/ijms262210858

**Published:** 2025-11-08

**Authors:** Isabela Siuffi Bergamasco, Nicolle Rakanidis Machado, Lais Alves do Nascimento, Beatriz Oliveira Fagundes, Fabio da Ressureição Sgnotto, Jorge Casseb, Sabri Saeed Sanabani, Luiz Henrique Da Silva Nali, Denis Miyashiro, José Antonio Sanches, Jefferson Russo Victor

**Affiliations:** 1 Post Graduation Program in Health Sciences, Santo Amaro University (UNISA), Sao Paulo 04743-030, Brazil; 2 Laboratory of Medical Investigation LIM-56, Division of Dermatology, Medical School, University of Sao Paulo, Sao Paulo 05403-000, Brazil; 3 Laboratory of Medical Investigation LIM-03, Clinics Hospital, Medical School, University of Sao Paulo, Sao Paulo 05403-010, Brazil; 4 Division of Dermatology, Medical School, University of Sao Paulo, Sao Paulo 05403-010, Brazil

**Keywords:** HAM/TSP, ATLL, HTLV-1, IgG, autoantibodies

## Abstract

Human T-lymphotropic virus type 1 (HTLV-1) infection is associated with a spectrum of clinical outcomes, ranging from lifelong asymptomatic carriage to severe conditions such as HTLV-1-associated myelopathy/tropical spastic paraparesis (HAM/TSP) and adult T-cell leukemia/lymphoma (ATLL). Although antibody responses are known to shape immune regulation, the functional relevance of IgG idiotype repertoires in HTLV-1 pathogenesis remains poorly understood. This study investigated the immunomodulatory effects of IgG from individuals with distinct HTLV-1 clinical outcomes. IgG was purified from pooled serum samples of asymptomatic carriers (ACs), HAM/TSP, and ATLL patients and used to stimulate peripheral blood mononuclear cells (PBMCs) from healthy donors. Cytokine production in CD4^+^, CD8^+^, and γδ T cells was assessed by flow cytometry. Additionally, proteome-wide IgG reactivity was evaluated using a human protein microarray encompassing over 21,000 proteins, and bioinformatic analyses were conducted to identify protein–protein interaction networks and tissue-specific autoreactivity. HAM/TSP-derived IgG selectively enhanced IFN-γ production in all T-cell subsets and suppressed IL-4 in CD4^+^ T cells. ATLL-derived IgG induced IL-9 and IL-13 production in CD4^+^ T cells, and both HAM/TSP and ATLL IgG elevated IL-13 levels in CD8^+^ T cells. Microarray data revealed distinct autoreactive IgG profiles across clinical groups, targeting immune-related proteins, apoptotic regulators, and proteins expressed in T cells, monocytes, and non-immune tissues such as brain and testis. Notably, no functional or structural clustering was observed in protein–protein interaction networks, suggesting these reactivities reflect complex, idiotype-specific immune alterations rather than compensatory responses. The present findings suggest that HTLV-1 infection may be associated with the development of distinct IgG repertoires that potentially modulate cytokine responses and exhibit broad reactivity toward human proteins. Such patterns could contribute to immune dysregulation and may partially explain the divergent clinical trajectories observed in HAM/TSP and ATLL. Further investigations are warranted to validate these observations at the individual level and to clarify their mechanistic relevance in disease progression.

## 1. Introduction

Human T-lymphotropic virus type 1 (HTLV-1) is a retrovirus implicated in a range of inflammatory conditions, including pulmonary alveolitis, chronic dermatitis, arthropathy, uveitis, conjunctivitis, and interstitial keratitis [1,2,3,4,5] but 1 to 5% of these individuals go on to develop ATLL or HAM/TSP [6,7,8,9]. In recent years, the discourse surrounding the mechanisms that mediate differentially induced or naturally developed IgG repertoires has intensified [10,11,12]. However, there remains a lack of robust evidence to elucidate the molecular underpinnings of the hypothesis known as “the hooks without bait” [13]. This hypothesis proposes that the IgG idiotypes induced by human exposure to environmental antigens, genetic predisposition, and infections may result in the generation of distinct IgG idiotype sets. These sets can facilitate unforeseen interactions with clonal and conserved molecules expressed on the membranes of lymphocytes and other immune-system cells, ultimately leading to immune modulation. It is posited that these IgG-lymphocyte interactions may yield either regulatory or inflammatory effects, with the potential to influence the overall immune response. Understanding this intricate network could pave the way for advancements in pathogenesis comprehension and therapeutic development.

Several studies have reported that different IgG idiotype repertoires can independently mediate immune modulation in human lymphocytes [14,15,16], including the cytokine production of thymic and peripheral αβT, γδT, and B cells, contingent upon the immune status of the donors. For instance, research has shown that IgG from patients with Atopic Dermatitis influences IL-17, IL-22 and IL-10 production by T cells [17,18], IgG from non-atopic donors can mediate the regulation of IL-17, IFN-γ and IL-10 production by T cells [19], and IgG from both HIV-1-exposed non-infected and infected individuals can modulate IFN-γ production by T and B cells [20]. In the context of B cells, recent findings indicate that human IgG from non-atopic individuals can induce IL-10-producing B cells (B10 cells) in both the infant thymus and adult PBMCs [21].

In the realm of HTLV-1-infected patients, it has been established that controlling HTLV-1 proliferation in infected individuals relies on the induction of effector immune mechanisms, including the production of IgG antibodies [22]. The immunological stimulation triggered by HTLV-1 infection, particularly the IgG-mediated humoral response, may play a critical role in shaping the clinical progression of individuals with lifelong asymptomatic infection, HAM/TSP, or ATLL. Furthermore, an association has been identified between the development of HAM/TSP and elevated levels of HTLV-1 antibodies [23], suggesting that the intensity and/or specificity of the humoral response may be related to the progression of HTLV-1 infection and the manifestation of associated inflammatory diseases.

Administration of polyvalent IgG preparations pooled from thousands of healthy individuals has shown therapeutic benefit across various immune-mediated conditions. Notably, such treatment has been effective in preventing recurrent spontaneous miscarriage [24], a condition frequently associated with the presence of autoantibodies [25,26,27], as well as in severe pediatric asthma [28], atopic dermatitis (AD), where marked clinical improvement has been documented [29,30], and pemphigus vulgaris, in which cases of complete remission have been reported [31]. Despite these encouraging observations emphasizing the immunoregulatory capacity of polyvalent IgG, the molecular determinants that drive these effects and the potential condition-specific properties of IgG preparations originating from donors with active immune responses or infections remain to be fully elucidated [32,33,34,35].

More recently, it has been shown that IgG from HAM/TSP patients can, in vitro, increase the production of IL-17 by CD4^+^ T cells, decrease the frequency of IL-4-producing CD4^+^ T cells, enhance IFN-γ production by CD8^+^ T cells, and reduce IL-4 production by CD8^+^ T cells. Additionally, the same study indicated that IgG from ATLL patients can decrease the frequency of IL-4-producing CD4^+^ T cells, reduce the frequency of IFN-γ-producing γδT cells, and lower the frequency of IL-10-producing B cells. Both HAM/TSP and ATLL patient IgG were also found to decrease the frequency of IFN-γ-producing B cells [36].

These findings, particularly the recent investigations into IgG from HTLV-1-infected patients, underscore a broad spectrum of IgG-mediated regulation of immune functions. To further investigate these findings, it is crucial to examine additional cellular effects that may contribute to peripheral cytokine-mediated immunoregulation, as well as to elucidate the potential interactions between the IgG repertoire—shaped by the distinct clinical manifestations of HTLV-1 infection—and self-proteins expressed in the human immune system and other tissues. Such analyses may clarify the specific mechanisms, interactions, and immunomodulatory roles of IgG in this context.

Building upon these observations, the present study aims to evaluate the effects of purified IgG on cytokine production by healthy peripheral T cells, including CD4^+^, CD8^+^, and TCRγδ^+^ subsets, and to characterize the idiotype repertoire associated with each of the three major clinical outcomes of HTLV-1 infection. To this end, we conducted in vitro experiments in which distinct formulations of purified IgG were cultured with peripheral blood mononuclear cells (PBMCs) from healthy donors. In parallel, we employed a comprehensive human proteome microarray containing over 21,000 individual human proteins—including various isoforms and protein fragments—representing 16,794 unique genes. This platform encompasses more than 84% of all human proteins categorized within key functional classes, as defined by the Human Protein Atlas (www.proteinatlas.org (accessed on 22 September 2025)).

## 2. Results

### 2.1. Differential Effects of IgG from ACs, HAM/TSP, and ATLL Patients on Cytokine Production by Healthy CD4^+^, CD8^+^, and γδ T Cells

To assess the immunomodulatory properties of IgG idiotype repertoires associated with distinct clinical outcomes of HTLV-1 infection, we purified and pooled IgG from asymptomatic carriers (ACs), patients with HAM/TSP, and individuals with ATLL. These IgG preparations were used to stimulate peripheral blood mononuclear cells (PBMCs) derived from healthy, HTLV-1–uninfected donors. IgG from healthy controls (HCs) was included as a comparator in all assays; PBMC donors were independent from the serum donors used for IgG purification.

**Figure 1:**
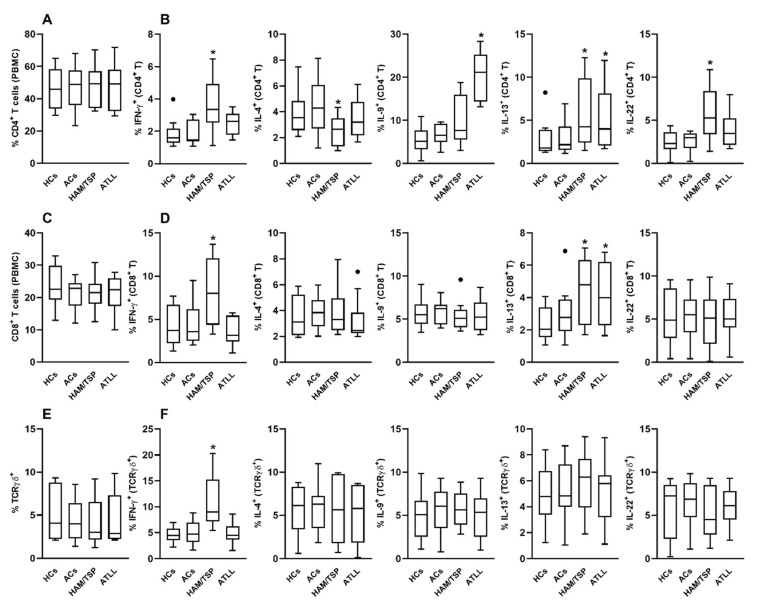
Modulatory Effects of IgG from HTLV-1-Infected Patients on Cytokine Production by Healthy Peripheral CD4^+^, CD8^+^, and TCRγδ^+^ T Cells. Peripheral blood mononuclear cells (PBMCs) from healthy donors (*n* = 10) were cultured for 3 days with 100 μg/mL of purified IgG obtained from healthy controls (HCs), HTLV-1-infected asymptomatic carriers (ACs), patients with HAM/TSP, or patients with ATLL. Following culture, viable CD4^+^, CD8^+^, and TCRγδ^+^ T cells were assessed by flow cytometry for their frequency (**A**,**C**,**E**) and intracellular cytokine production (**B**,**D**,**F**), including IFN-γ, IL-4, IL-9, IL-13, and IL-22. Data are presented as box-and-whisker plots indicating minimum, maximum, and interquartile ranges. Symbols represent individual data points, with outliers identified using the Tukey method. * denotes statistical significance (*p* ≤ 0.05) compared to the HCs IgG condition. ● represents outlier values.

After three days of culture, the frequency of CD4^+^ T cells remained unchanged across all experimental conditions (Figure 1A). However, analysis of cytokine production revealed distinct patterns. IgG from HAM/TSP patients significantly increased the frequency of IFN-γ–producing CD4^+^ T cells compared to the HC IgG condition (Figure 1B). Conversely, the same HAM/TSP-derived IgG reduced the frequency of IL-4–producing CD4^+^ T cells. IgG from ATLL patients induced a significant increase in IL-9–producing CD4^+^ T cells compared to all other groups. Additionally, both HAM/TSP and ATLL-derived IgG enhanced IL-13 production by CD4^+^ T cells relative to the HC condition. Only the HAM/TSP group showed a significant induction of IL-22–producing CD4^+^ T cells (Figure 1B).

In the CD8^+^ T cell compartment, no significant differences in cell frequency were observed between groups (Figure 1C). Nevertheless, functional differences emerged. IgG from HAM/TSP patients significantly elevated the frequency of IFN-γ–producing CD8^+^ T cells compared to the HC group (Figure 1D). Moreover, both HAM/TSP- and ATLL-derived IgG increased IL-13 production by CD8^+^ T cells. No significant effects were observed on IL-4, IL-9, or IL-22 production in CD8^+^ T cells under any condition (Figure 1D).

Analysis of γδ T cells similarly revealed no differences in cell frequency between culture conditions (Figure 1E). However, HAM/TSP-derived IgG significantly enhanced the proportion of IFN-γ–producing γδ T cells compared to the HC group (Figure 1F). No notable effects were observed on IL-4, IL-9, IL-13, or IL-22 production by γδ T cells in any of the tested conditions (Figure 1F).

### 2.2. HTLV-1-Infected Patients Exhibit Distinct IgG Profiles Targeting Immune System Proteins

We analyzed 400 major human proteins associated with the immune system, categorized into cytokines, cytokine receptors, antibodies, antibody receptors, clusters of differentiation (CDs), apoptosis-related molecules, and complement system proteins. Details regarding the number of evaluated proteins in each category, the potential targets for each IgG donor group, and the threshold calculations are provided in Supplementary Table S1.

**Figure 2:**
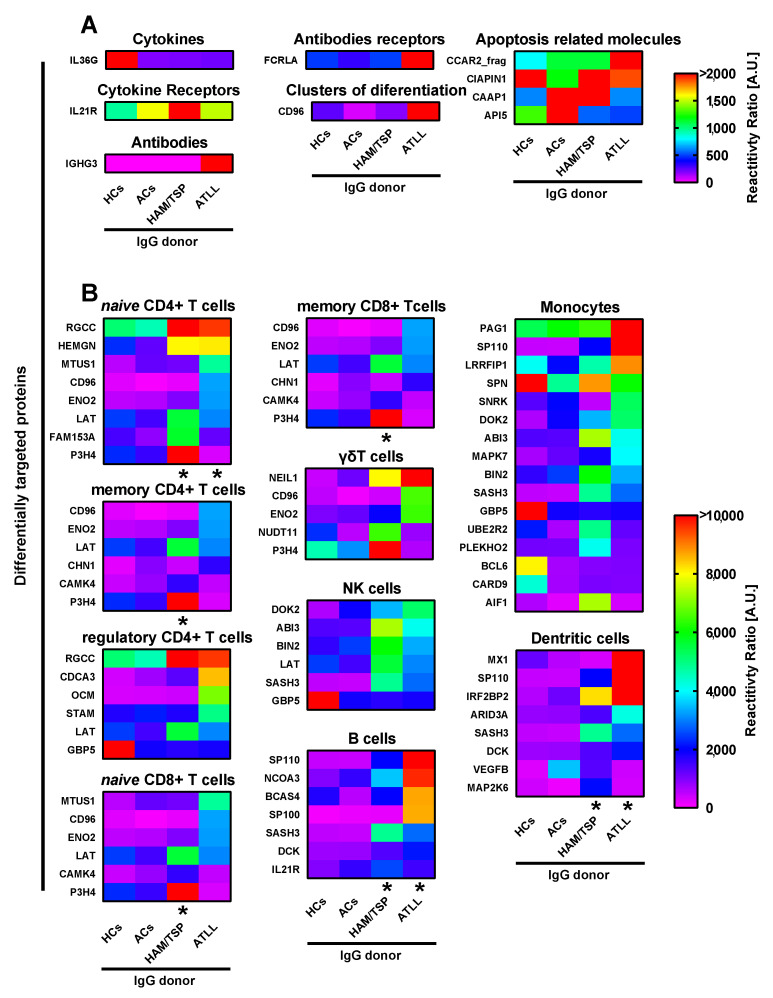
Targeting of immune component and immune cell proteins by IgG from different HTLV-1 donor groups. Human proteome microarray profiling was performed using HuProt™ Human Proteome Microarrays (CDI Labs, Puerto Rico) with pooled serum samples from four groups: 40 healthy controls (HCs), 14 asymptomatic carriers (ACs), 16 patients with HAM/TSP, and 11 patients with ATLL. Panel (**A**) illustrates immune components, while Panel (**B**) lists immune cells. Heatmaps display proteins identified as targets based on their reactivity intensity, with a ratio greater than 2 compared to the HCs threshold value. * = FDR < 0.05 compared with the respective HCs IgG row.

As shown in Figure 2A, elevated IgG reactivity against interleukin 36 gamma (IL36G) was observed exclusively in healthy controls. Among the 84 evaluated cytokine receptors, HAM/TSP donors exhibited strong IgG reactivity against interleukin 21 receptor (IL21R). In the antibody and antibody receptor groups (16 and 18 proteins, respectively), IgG from ATLL donors uniquely targeted immunoglobulin heavy constant gamma 3 (IGHG3) and Fc receptor-like A (FCRLA).

In the CD group (85 proteins), IgG from ATLL donors displayed strong recognition of the CD96 molecule. For apoptosis-related proteins (41 proteins), IgG from ATLL donors strongly recognized cell cycle and apoptosis regulator 2 (CCAR2_frag), while HAM/TSP donors targeted cytokine-induced apoptosis inhibitor 1 (CIAPIN1) and caspase activity and apoptosis inhibitor 1 (CAAP1). Conversely, IgG from ACs donors demonstrated strong reactivity toward apoptosis inhibitor 5 (API5). No significant IgG recognition of complement system proteins was detected in any group.

### 2.3. HTLV-1-Infected Patients Produce IgG Targeting Immune Cells

We extended our analysis to 1224 proteins associated with naïve and memory CD4^+^ and CD8^+^ T cells, regulatory CD4^+^ T cells, γδ T cells, natural killer (NK) cells, B cells, monocytes, and dendritic cells (DCs) (see Supplementary File S1 for details).

As demonstrated in Figure 2B, for naïve CD4+ T cells, HAM/TSP donors’ IgG recognized regulator of cell cycle (RGCC), hemogen (HEMGN), linker for activation of T cells (LAT), family with sequence similarity 153 member A (FAM153A), and prolyl 3-hydroxylase family member 4 (P3H4). In contrast, ATLL donors’ IgG targeted microtubule-associated scaffold protein 1 (MTUS1), CD96, and enolase 2 (ENO2). Memory CD4^+^ T cells showed overlapping recognition patterns, with HAM/TSP IgG targeting LAT and P3H4 and ATLL IgG targeting CD96 and ENO2. Additionally, ATLL donors’ IgG recognized chimerin 1 (CHN1), while HAM/TSP donors’ IgG targeted calcium/calmodulin-dependent protein kinase IV (CAMK4).

In regulatory CD4^+^ T cells, RGCC was a common target of HAM/TSP and ATLL donors’ IgG, while ATLL IgG also strongly recognized cell division cycle-associated 3 (CDCA3), oncomodulin (OCM), and signal transducing adaptor molecule (STAM). IgG from healthy controls strongly recognized guanylate-binding protein 5 (GBP5), a reactivity absent in HTLV-1-infected groups (Figure 2B).

Similar recognition patterns were observed for *naïve* and memory CD8^+^ T cells, where shared targets included CD96, ENO2, LAT, CHN1, CAMK4, and P3H4. Additionally, γδ T cells were identified as potential targets based on shared protein recognition patterns, including unique targeting of nei-like DNA glycosylase 1 (NEIL1) by HAM/TSP and ATLL donors and nudix hydrolase 11 (NUDT11) exclusively by HAM/TSP donors (Figure 2B).

For NK cells, overlapping recognition of LAT and GBP5 was observed, alongside unique targets such as docking protein 2 (DOK2), ABI family member 3 (ABI3), bridging integrator 2 (BIN2), and SAM and SH3 domain-containing 3 (SASH3). SASH3 was also targeted by HAM/TSP and ATLL IgG in B cells, with additional unique targets such as SP110 nuclear body protein (SP110) and nuclear receptor coactivator 3 (NCOA3) identified in ATLL donors (Figure 2B).

In monocytes, eight additional proteins were targeted by HTLV-1-infected donors, and two proteins were uniquely recognized by IgG from healthy controls. In DCs, recognition profiles were generally mild, except for strong reactivity toward MX dynamin-like GTPase 1 (MX1) by ATLL donors and interferon regulatory factor 2-binding protein 2 (IRF2BP2) by both HAM/TSP and ATLL donors (Figure 2B).

### 2.4. HTLV-1-Infected Patients Produce IgG Targeting Various Human Tissues

**Figure 3:**
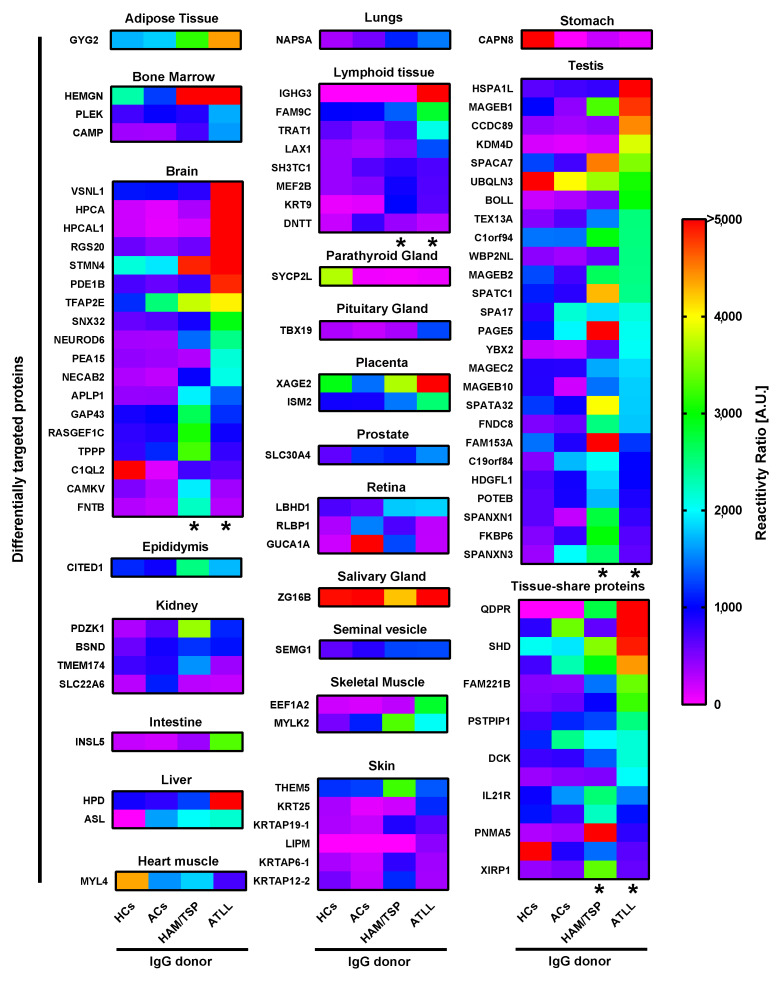
Targeting of specific and shared proteins expressed in major human tissues by IgG from different HTLV-1 donor groups. Human proteome microarray profiling was performed using HuProt™ Human Proteome Microarrays with pooled serum samples from four groups: 40 healthy controls (HCs), 14 asymptomatic carriers (ACs), 16 patients with HAM/TSP, and 11 patients with ATLL. Tissue-specific proteins are listed in alphabetical order from the upper left panel to the lower right panel, while tissue-shared proteins are displayed in the lowest right panel. Heatmaps show proteins identified as targets based on their reactivity intensity, with a ratio greater than 2 compared to the HCs threshold value. * = FDR < 0.05 compared with the respective HCs IgG row.

We investigated whether the IgG repertoire generated in response to HTLV-1 infection, across different pathological manifestations, could recognize unexpected proteins in human tissues. A total of 2214 proteins, associated with 32 human tissues based on enriched gene expression, were evaluated. Of these, 22 tissues exhibited at least one protein as a potential IgG target from one or more donor groups. Due to the diversity of tissue-related IgG targets, we presented all identified proteins in alphabetical order in Figure 3.

Among the targeted proteins, IgG from healthy controls (HCs) uniquely and intensely recognized myosin light chain 4 (MYL4, heart muscle), synaptonemal complex protein 2-like (SYCP2L, parathyroid gland), calpain 8 (CAPN8, stomach), and ubiquilin 3 (UBQLN3, testis).

In ACs, the most prominent and unique IgG reactivity was directed toward guanylate cyclase activator 1A (GUCA1A), a retina-specific protein.

IgG from HAM/TSP donors displayed intense reactivity against cbp/p300-interacting transactivator with Glu/Asp-rich carboxy-terminal domain 1 (CITED1, epididymis), PDZK1-interacting protein 1 (PDZK1IP1, kidney), myosin light chain kinase 2 (MYLK2, skeletal muscle), and thioesterase superfamily member 5 (THM5, skin), among others, although some were recognized with lower intensity.

The most diverse and distinct tissue-specific IgG profile was observed in ATLL donors. These IgG samples strongly targeted glycogenin 2 (GYG2, adipose tissue), visinin-like 1 (VSNL1, brain), hippocalcin (HPCA, brain), hippocalcin-like 1 (HPCAL1, brain), regulator of G protein signaling 20 (RGS20, brain), insulin-like 5 (INSL5, intestine), 4-hydroxyphenylpyruvate dioxygenase (HPD, liver), napsin A aspartic peptidase (NAPSA, lungs), and immunoglobulin heavy constant gamma 3 (IGHG3, lymphoid tissue). Several additional proteins were recognized with less intensity or not exclusively by ATLL IgG donors.

### 2.5. Evaluation of Protein–Protein Interaction Network (PPIN) and Functional Enrichment Analysis Among Targeted Proteins

Following the evaluation of all protein groups across the samples, we analyzed the PPINs among the identified targets. Each protein group was examined individually to identify known and predicted interactions, including those derived from curated databases, experimentally determined associations, and predictions based on gene neighborhood, gene fusions, and gene co-occurrence. Additional sources of evidence, such as text mining, co-expression, and protein homology, were also considered. Functional enrichment analyses were performed using Gene Ontology, COMPARTMENTS, WikiPathways, Reactome, and TISSUES databases.

**Figure 4:**
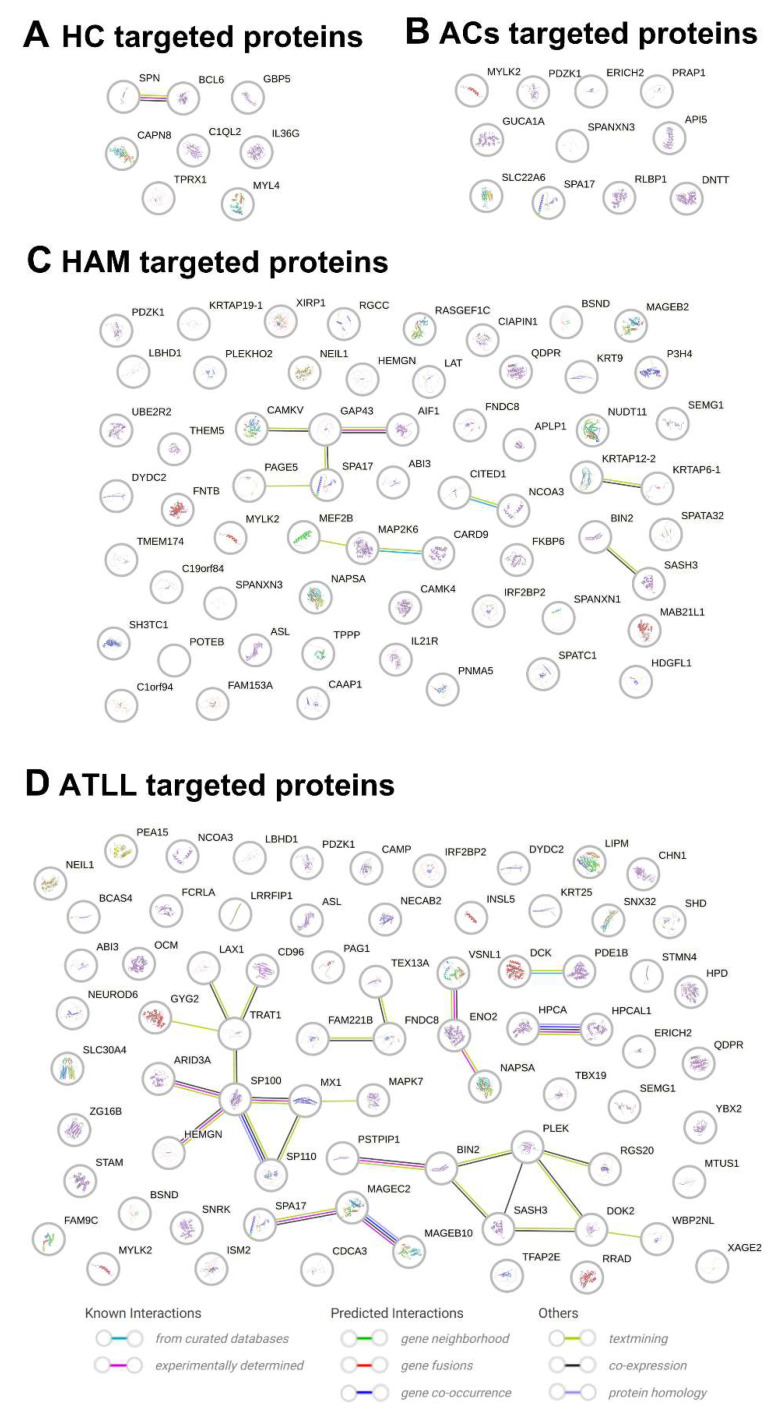
Protein–Protein Interaction Network (PPIN) Analysis of Targeted Proteins in IgG from Different HTLV-1 Donor Groups. PPINs were generated for proteins targeted by IgG in 40 healthy controls (HCs, (**A**)), 14 asymptomatic carriers (ACs, (**B**)), 16 patients with HAM/TSP (HAM, (**C**)), and 11 patients with ATLL (ATLL, (**D**)) using the STRING platform (STRING Consortium, v12.0, 2024). Known interactions, predicted interactions, and additional evidence types were independently assessed for all proteins identified within each group. Each node represents a targeted protein, with those of known structure displayed together with their respective structural representations within the nodes.

In the healthy control (HC) group, eight targeted proteins were identified, revealing evidence of experimentally determined, text-mined, and co-expression-based interactions between two proteins (Figure 4A). In the asymptomatic carrier (AC) group, no interactions were detected among the 11 targeted proteins (Figure 4B).

The HAM group (61 targeted proteins) exhibited limited connectivity, with a low frequency of experimentally validated and curated database interactions. A few text-mined associations and only four co-expression relationships were observed, while no evidence of protein homology was detected (Figure 4C). The ATLL group (73 targeted proteins) showed a similar pattern to the HAM group but with slightly greater co-expression, including five small clusters of interacting proteins and homology detected between three protein pairs (Figure 4D).

## 3. Discussion

In the present study, we further demonstrated a prominent immunomodulatory role for IgG, particularly from HAM/TSP patients, which significantly enhanced IFN-γ production across all three major T-cell subsets evaluated—CD4^+^, CD8^+^, and γδ T cells.

These findings align with previous transcriptomic analyses showing that a subset of interferon-stimulated genes is overexpressed in HAM/TSP patients but not in asymptomatic carriers [37]. Moreover, prior evidence has linked the infiltration of HTLV-1–specific CD8^+^ T cells to HAM/TSP pathogenesis through bystander neural damage involving the apoptosis of oligodendrocytes in proximity to virus-infected CD4^+^ T cells [38]. Our observation that only HAM/TSP-derived IgG enhances IFN-γ production in CD8^+^ T cells supports this proposed mechanism and highlights a possible contribution of IgG to disease progression. Additionally, a known association between IFN-γ gene polymorphisms and elevated IFN-γ plasma levels in HTLV-1–infected individuals suggests that early, robust IFN-γ responses may act as a trigger for HTLV-1–associated symptoms [39]. In this context, our findings raise the hypothesis that IgG antibodies could contribute to the initiation or amplification of IFN-γ–mediated responses, potentially facilitating the development of HAM/TSP—a possibility warranting further in vivo investigation.

Regarding IL-4, we found that HAM/TSP-derived IgG reduced its production in CD4^+^ T cells, the primary cellular source of this cytokine. This observation is noteworthy given that IL-4 has been reported to promote leukemic cell proliferation in vitro [40,41]. However, peripheral IL-4 levels in HTLV-1–infected individuals have not consistently correlated with HAM/TSP development [42,43], suggesting that the IgG-mediated suppression of IL-4 observed here may not be a major contributor to HAM/TSP pathogenesis.

A novel finding from our study is that IgG from ATLL patients significantly increased IL-9 production in CD4^+^ T cells from healthy donors. While previous studies have shown that ATLL-derived leukemic cells can produce IL-9 autonomously [44], our results suggest that the IgG repertoire from ATLL patients may also promote IL-9 expression in non-infected immune cells. Although Th9 cells have been implicated in modulating neurological inflammation and may act as protective players in HAM/TSP [45], our findings indicate that IL-9 induction by IgG is specific to ATLL-derived antibodies. This suggests that IgG from HAM/TSP patients does not contribute to Th9-mediated protective mechanisms.

Another novel aspect of our study is the observation that IgG from both HAM/TSP and ATLL patients can induce IL-13 production not only in CD4^+^ but also in CD8^+^ T cells. Previous studies have shown that HTLV-1–transformed cells can secrete IL-13 in vitro [46] and that IL-13 may support viral persistence and leukemic transformation via autocrine stimulation of HTLV-1–infected cells. Our results suggest that, in individuals predisposed to HAM/TSP or ATLL, the IgG repertoire itself may contribute to the amplification of IL-13–mediated signaling, possibly creating a microenvironment conducive to viral persistence and immune dysregulation.

To explore potential mechanisms underlying these IgG-mediated effects, we further investigated possible interactions between IgG and human self-proteins relevant to HTLV-1 pathogenesis. Our goal was to address a gap in the literature, as recent reviews have noted a lack of comprehensive studies examining the role of antibodies in the clinical manifestations of HTLV-1 infection [47]. By linking specific IgG idiotype repertoires to functional outcomes in T-cell subsets, our findings contribute to a growing body of evidence suggesting that IgG antibodies may play an active role in shaping disease trajectories in HTLV-1–infected individuals.

In the context of human cytokines, our findings reveal an unprecedented observation that healthy individuals produce anti-IL-36 IgG, but this reactivity is lost in HTLV-1-infected patients. IL-36, a member of the IL-1 superfamily, plays a critical role in initiating inflammatory responses at epithelial barriers, including the skin, lungs, and gut. It exerts potent effects on immune cells, modulating responses to danger- and pathogen-associated molecular patterns, though its expression is influenced by exogenous pathogens, including viruses [48]. Our results suggest that healthy individuals may regulate soluble IL-36 through anti-IL-36 IgG, a mechanism potentially disrupted in HTLV-1 infection, which could lead to inflammatory effects at epithelial barriers.

Another notable cytokine-related observation is the strong recognition of the IL-21 receptor (IL-21R) by IgG from HAM/TSP patients. IL-21 is critical for the survival and cytotoxicity of CD8^+^ T cells and plays an essential role in controlling chronic viral infections [49]. Increased IL-21 expression has been linked to the activation of antiviral responses, while decreased expression may reflect viral inhibition of IL-21 to facilitate HTLV-1 dissemination and HAM/TSP development [50]. Additionally, ATLL leukemic cells overexpress IL-21R, contributing to ATLL pathophysiology [51]. However, our findings indicate that HAM/TSP patients produce anti-IL-21R IgG, a phenomenon not observed in other groups, including ATLL patients. This could hypothetically block IL-21 binding to its receptor, thereby impairing CD8^+^ T cell activation and exacerbating HTLV-1 dissemination and HAM/TSP development. Further investigations are required to elucidate this mechanism.

IgG from ATLL patients also showed strong reactivity against CD96, a molecule involved in T cell activation. Anti-CD96 antibodies have been shown to directly stimulate T cell proliferation, cytokine secretion, and resistance to Treg suppression [52]. However, in ATLL patients, the production of anti-CD96 IgG may fail to induce antitumor activity or may even inhibit T cell activation, potentially contributing to the severity of ATLL.

These findings indicate that HTLV-1-infected individuals can develop distinct profiles of immune-modulating autoantibodies, which, in combination, may contribute to the varying patterns of immune control and clinical manifestations observed across different stages or forms of HTLV-1 infection.

When examining proteins overexpressed in major immune cell populations, we found that HAM/TSP IgG frequently targeted P3H4, a nucleolar protein associated with bladder cancer [53], across CD4^+^, CD8^+^, and γδ T cell populations. Despite no literature supporting a functional role for P3H4 in these cells, its recognition warrants further study. Similarly, ENO2 (neuron-specific enolase), primarily involved in glycolysis in neuronal cells, was recognized by ATLL IgG in multiple T cell subsets. While ENO2 is not typically associated with immune cells, its recognition may suggest a link between HTLV-1 infection and neurological pathways [54].

The RGCC protein, a regulator of the cell cycle and a potential contributor to leukemia [55], was also targeted by IgG from both HAM/TSP and ATLL patients. This suggests abnormal expression of RGCC in HTLV-1 infection, potentially triggering specific IgG responses that merit further investigation.

In NK cells the DOK2 protein, associated with NK cell inhibition [56], was intensely targeted by IgG from HAM/TSP and ATLL patients. Since DOK2 also influences T cell activation via the T cell receptor [57], anti-DOK2 IgG production may impact immune regulation and requires additional exploration.

Regarding B cells, ATLL IgG strongly recognized proteins like SP110 and SP100, which are linked to B cell survival [58], NCOA3, associated with leukemia development [59], and BCAS4, related to cancer progression [60]. These findings suggest potential interactions between ATLL IgG and B cells that could influence disease outcomes.

Although fewer monocyte-related proteins were evaluated, we identified 16 as targets, particularly for HAM/TSP and ATLL IgG. Proteins such as LRRFIP1, involved in monocyte activation and lysosomal function [61], and PAG1, linked to allergy development [62], were prominently recognized. IgG targeting of these proteins may indicate a role in immune modulation, although further studies are needed. The production of anti-LRRFIP1 IgG may suggest overexpression of this protein as part of an antiviral response. However, its precise effects, mediated by direct IgG-protein interactions, remain to be elucidated. Additionally, IgG from HAM/TSP donors showed intense and exclusive recognition of UBE2R2 and PLEKHO2 proteins. UBE2R2 expression has been linked to brain tumors [63], and its targeting by HAM/TSP IgG may be associated with the neurological disorders observed in these patients. PLEKHO2, on the other hand, is related to macrophage survival [64]. The role of anti-PLEKHO2 IgG warrants further investigation, as it may influence monocyte functions in the context of an antiviral response.

For DCs, MX1 stood out as a target, especially in ATLL patients. MX1, a viral restriction factor, is typically upregulated by interferons but is downregulated in HTLV-1-positive cells [65]. Anti-MX1 IgG in ATLL patients may represent an additional mechanism influencing antiviral responses.

Similarly to the findings observed when investigating immune system-related molecules, an overall analysis of proteins associated with immune cells revealed that certain autoantibodies may be lost following HTLV-1 infection. This loss could potentially reduce the frequency of interactions with key immune cells such as CD4^+^ T cells, NK cells, and monocytes—particularly those interactions mediated by SPN and GBP5 proteins. Conversely, several other proteins became IgG targets only after infection.

Notably, in the context of immune cell–associated proteins, a greater number of targets were identified, particularly among individuals with clinical manifestations of HTLV-1 infection (i.e., HAM/TSP and ATLL). The statistical relevance of these targets suggests a stronger propensity for IgG reactivity toward proteins expressed in CD4^+^ T cells, CD8^+^ T cells, B cells, and dendritic cells. This suggests that these groups develop a broader or more specific repertoire of IgG autoantibodies capable of mediating interactions with immune cells. Such interactions may trigger downstream effects that remain to be fully elucidated but are likely to differ between the HAM/TSP and ATLL groups. These findings point toward a potential role of differential IgG-cell interactions in shaping the distinct cellular immune responses observed in these clinical forms of HTLV-1 infection.

Finally, our findings highlight the complexity of IgG targeting in non-immune tissues, particularly the brain and testis. In the brain, at least seven proteins were exclusively targeted by IgG from ATLL patients, while six were exclusively targeted by IgG from HAM/TSP patients. This finding is significant, as HAM/TSP patients experience neurological disturbances distinct from the leukemia or lymphoma observed in ATLL patients. Moreover, the coexistence of these disorders is rare [66]. It is plausible that the distinct sets of IgG idiotypes produced by individuals predisposed to each clinical form may contribute to their respective manifestations.

In the testis, we identified 25 proteins as targets recognized by IgG from both HAM/TSP and ATLL patients, with some also targeted by ACs. This high level of complexity prevents the pinpointing of specific proteins as key targets. Instead, it suggests that the testis might be targeted by diverse IgG idiotypes that are challenging to characterize. Such complexity could hinder our understanding of the inflammatory effects of HTLV-1 infection on testicular tissue and may explain prior observations of autoantibodies against testis antigens in HTLV-1-infected individuals without clear differences across clinical statuses [67].

These observations prompt further consideration of broader immune-mediated mechanisms potentially relevant to HTLV-1 pathogenesis. The broader implications of these findings may also intersect with established paradigms of autoimmunity in cancer and neuroinflammation. For instance, the autoimmune features observed in HAM/TSP share conceptual similarities with paraneoplastic neurological syndromes (PNS), in which immune responses—often antibody-mediated—target neural antigens due to molecular mimicry or ectopic expression in malignancies [68]. Although HAM/TSP is not typically classified as a PNS, the presence of IgG reactivity against brain-specific proteins in both HAM/TSP and ATLL patients raises the hypothesis that onconeural-like autoimmunity may be involved in HTLV-1–associated neurodegeneration. In parallel, the detection of ATLL IgG targeting multiple proteins previously associated with tumorigenesis and immune evasion, including SP110 [58], NCOA3 [69], and ENO2 [54], suggests potential cross-reactivity with tumor-associated antigens (TAAs). Such cross-reactivity might reflect either an anti-tumor response or an epiphenomenon of dysregulated humoral immunity in ATLL. While speculative, these parallels warrant further investigation, particularly in light of recent studies linking cancer immunity, autoantibody production, and systemic immune dysregulation [70,71].

Given the extensive repertoire of proteins targeted by IgG—particularly in HTLV-1–infected individuals—our comprehensive analysis of tissue-associated proteins suggests that the HTLV-1–induced immune response may give rise to novel IgG idiotypes capable of recognizing antigens not previously associated with this infection. These antibodies may interact with a variety of tissues, thereby contributing to the distinct immunological profiles observed across different clinical manifestations. For example, in epithelial, glandular, or pulmonary tissues, autoreactive antibodies could engage local proteins, modulating their function with clinically relevant systemic consequences that may be specific to each clinical form of HTLV-1 infection. Although we were unable to demonstrate a direct causal relationship between these interactions and the resulting pathophysiological outcomes, our findings provide a foundation for future studies investigating the complex interplay between HTLV-1 infection, IgG idiotype diversity, and immune regulation in human tissues.

Through PPIN analysis, we broadly examined the network of targeted proteins in each group, yielding valuable insights. Structural homology analysis revealed that the recognized proteins did not share structural similarities, with the sole exception occurring in the ATLL group. This finding suggests that the repertoire of autoreactive IgG idiotypes—ranging from healthy individuals to HTLV-1-infected individuals at different clinical stages—did not arise from the induction of autoreactive IgG by a small subset of proteins capable of eliciting widespread cross-reactivity. Furthermore, it implies that the acquired IgG repertoire, particularly in the HAM/TSP and ATLL groups, may mediate diverse and specific recognition of the identified human proteins.

Another aspect of our in silico analysis was the investigation of significant associations between the targeted proteins and various biological attributes, including biological processes, molecular functions, local network clusters, Reactome pathways, WikiPathways, disease-gene associations, annotated keywords, protein domains and features, subcellular localization, and protein domains. However, no significant associations were identified for the targeted proteins in any of the HTLV-1-infected groups. This observation suggests that the set of HTLV-1-induced IgG idiotypes is not directly related to any biological processes engaged in the host response to infection, opening new avenues for future research.

These differences in protein recognition between HAM/TSP and ATLL patients may reflect distinct mechanisms underlying their respective clinical manifestations. However, the involvement of these proteins in disease pathogenesis remains speculative due to the limited scope of this study.

In summary, our findings suggest a complex network of IgG idiotypes induced by HTLV-1 infection, wherein distinct patterns of IgG autoantibodies are generated and differentially associated with specific clinical manifestations. This recognition network appears to underlie a broad reactivity toward human proteins. Despite the study’s limitations, including the absence of individual-level validation for specific targets, our results provide a novel perspective on the antibody-mediated mechanisms involved in HTLV-1 infection.

Notably, the absence of significant clustering by functional category or structural homology in protein–protein interaction network analyses suggests that these IgG responses are unlikely to represent conventional compensatory or homeostatic immune mechanisms. Rather, they may reflect a broader and less well-characterized pattern of antibody-mediated recognition, potentially shaped by chronic infection and immune perturbation.

## 4. Methods

### 4.1. Samples

Serum samples were obtained from a subset of patients within a larger cohort of 233 individuals infected with HTLV-1. This subset included asymptomatic carriers (ACs; *n* = 14; 12 males and 2 females; age: 52.7 ± 2.7 years; median proviral load: 13 copies/1000 PBMCs), patients with HTLV-1-associated myelopathy/tropical spastic paraparesis (HAM/TSP; *n* = 16; 10 males and 6 females; age: 57.4 ± 2.2 years; median proviral load: 162 copies/1000 PBMCs), and patients with adult T-cell leukemia/lymphoma (ATLL; *n* = 11; 7 males and 4 females; age: 48.4 ± 4.6 years; median proviral load: 502 copies/1000 PBMCs). In this study, all ATLL patients included presented with the leukemic form of the disease. All HTLV-1-positive participants were recruited from the HTLV-1 outpatient clinic at the University of São Paulo and the Institute of Infectious Diseases “Emilio Ribas.” Healthy controls (HCs; *n* = 40; 17 males and 23 females; mean age ± SE: 47.5 ± 2.5 years) were non-infected individuals confirmed negative for HTLV-1 at the time of blood donation.

For the purposes of this preliminary, exploratory study, pooled serum samples were prepared for each HTLV-1-infected group (HCs, ACs, HAM/TSP, and ATLL) to allow high-throughput screening of IgG reactivity across the full proteome using a single microarray per group. This approach was chosen to reduce costs and technical complexity in the context of an initial survey of autoantibody targets, and to identify group-level trends in IgG recognition patterns. Each pooled sample was prepared by combining equal volumes of serum from all individuals within the corresponding healthy or clinical subgroup. Data from healthy controls (HCs) were used to establish group-specific thresholds and standard deviations applied in subsequent analyses.

HTLV-1 infection was initially identified using the Murex HTLV I + II (Abbott/Murex, Wiesbaden, Germany) and Vironostika HTLV I/II (bioMérieux bv, Boxtel, The Netherlands) enzyme immunoassays and was confirmed through HTLV BLOT 2.4 (Genelabs Diagnostics, Science Park, Singapore). The clinical diagnosis of HAM/TSP was based on WHO criteria for HTLV-1-associated diseases, while ATLL was diagnosed based on serological evidence of HTLV-1 infection coupled with cytological or histological confirmation of T-cell malignancy.

### 4.2. IgG Purification

Serum samples were processed using the Melon Gel IgG Spin Purification Kit (Thermo Fisher Scientific, Waltham, MA, USA), following the manufacturer’s protocol. The resulting IgG was sterilized using 0.20 µm syringe filters (Corning, Darmstadt, Germany) and stored at −80 °C until use in cell culture assays. Protein concentration was determined with the Coomassie (Bradford, UK) Protein Assay Reagent (Pierce, Thermo Fisher, Waltham, MA, USA), also according to the manufacturer’s instructions. Purity was assessed by SDS-PAGE and consistently exceeded 95%, with the remaining protein contaminants exhibiting molecular weights below 10 kDa—suggesting the presence of small protein fragments rather than full-length biologically active proteins such as cytokines. ELISA analysis confirmed undetectable levels of IgA, IgM, and IgE in all preparations. Subclass distribution of IgG was also assessed by ELISA and found to be comparable across all IgG pools. This purification strategy demonstrated high efficiency in recovering idiotype-functional IgG molecules while minimizing the presence of immune complexes and aggregated forms [72]. To ensure biosafety, all serum samples were UV-irradiated and subjected to extended freezing to inactivate potential HTLV-I contamination.

### 4.3. PBMC Culture with Purified IgG

Peripheral blood mononuclear cells (PBMCs) were isolated from healthy donors and washed before being resuspended in RPMI 1640 medium supplemented with 10% FetalClone III (HyClone, Logan, UT, USA). Cell viability and counts were determined using trypan blue exclusion in a Neubauer chamber. A total of 1 × 10^6^ viable PBMCs were seeded into each well of a 96-well plate (Costar, Glendale, AZ, USA) and cultured in the presence of 100 µg/mL purified IgG from pooled serum (patient or control groups). Parallel wells included a mock-treated control and an additional control condition with 100 µg/mL of commercially available intravenous immunoglobulin (IVIg). Cultures were maintained for three days, with brefeldin A (1 µg/mL; Sigma, Jerusalem, Israel) added during the final 12 h. After incubation, cells were prepared for flow cytometric analysis.

### 4.4. Flow Cytometry

Flow cytometry protocols followed established methods previously published by our group [73,74]. PBMCs were transferred to cytometry tubes and stained for surface markers using 1 µg of each monoclonal antibody per tube (excluding unstained controls). Samples were incubated for 30 min at 4 °C in the dark, washed with PBS, centrifuged, and fixed in 1% formaldehyde for at least 10 min. Surface staining included antibodies against human CD3, TCRγδ, CD4, and CD8 (BD Pharmingen, San Diego, CA, USA). For intracellular cytokine staining, cells were permeabilized with 0.05% saponin in PBS and incubated at 4 °C for 30 min with fluorochrome-conjugated antibodies against IFN-γ, IL-4, IL-9, IL-13, and IL-22, or their respective isotype controls (BD Pharmingen, San Diego, CA, USA). After washing, samples were resuspended in PBS for acquisition.

Data acquisition was conducted using an LSRII Fortessa flow cytometer (BD Biosciences, Milpitas, CA, USA), collecting 500,000 lymphocyte-gated events per sample, based on size and granularity. Compensation was performed using BD CompBeads coated with the same antibodies used in staining procedures. Gating strategies employed isotype controls and Fluorescence Minus One (FMO) controls for accurate identification of cytokine-positive populations. T-cell subsets were defined as follows: CD3^+^TCRγδ^+^ cells were classified as γδ T cells; CD4^+^CD8^−^ cells as CD4^+^ T cells; and CD8^+^CD4^−^ cells as CD8^+^ T cells. Cell viability was assessed using the Live/Dead viability dye (PE-Texas Red, Thermo Fisher Scientific). FlowJo software v10.8 (Tree Star, Ashland, OR, USA) was used for data analysis.

### 4.5. Microarrays for IgG Target Identification

The profiling of serum IgG reactivity was conducted utilizing HuProt™ Human Proteome Microarrays v4.0 (CDI Labs, Mayagüez, Puerto Rico), which comprise 23,736 distinct unique human proteins expressed in yeast (Saccharomyces cerevisiae) as GST fusion proteins. A comprehensive list of the proteins analyzed, along with their abbreviations and the corresponding group, cell type, or tissue classification, can be found in Supplementary File S1. Each protein was purified and represented across 20 separate array blocks. The arrays also included control proteins, such as fluorescently labeled landmarks (Rhodamine+IgG647) for grid alignment and positive controls for the incubation assays. Washing procedures employed PBS (pH 7.4) with 0.05% Tween 20, where each washing step lasted 10 s and was repeated three times following each incubation period. Before the assay, the microarrays were blocked for 30 min with Rockland Blocking Buffer MB-070. The incubation buffer was prepared by adding 10% blocking buffer to the washing buffer. Serum samples were diluted to a 1:500 ratio in the incubation buffer and incubated at 4 °C for 16 h with orbital shaking at 140 rpm.

For detection purposes, the secondary antibody employed was goat anti-human IgG (Fc) DyLight680 (0.1 µg/mL), incubated for 45 min at room temperature in the incubation buffer. The scanning of the microarrays was performed using the Innopsys InnoScan 710-IR Microarray Scanner (Innopsys Inc., Sunnyvale, CA, USA), set at a resolution of 10 µm and a scanning gain of 30, with low laser power (680 nm, red).

Initially, the microarrays were probed with the secondary antibody following a 45-min incubation in blocking buffer to evaluate any background interactions that might affect the primary assay. Subsequently, the arrays were incubated with human sera, followed by staining with the secondary antibody and subsequent scanning. Spot intensities were quantified from 16-bit grayscale TIFF files, which offer a broader dynamic range than the microarray image files used in this study. Grid alignment and image quantification were performed using Mapix 9.1.0 (Innopsys Inc., Sunnyvale, CA, USA), with additional data processing carried out in R 4.3.2.

For each feature on the array, both median fluorescent foreground and local background intensities were recorded. Background-corrected spot intensities were calculated, with median foreground intensities averaged and variations among duplicate spots addressed. Background interactions from the secondary antibody were subtracted from the sample signals. Signal intensity ratios comparing the sample to the secondary antibody background were computed for each tested protein, excluding interactions with intensity ratios of ≤2 as non-specific. The remaining data were organized based on the corrected intensity ratio of the sample to the background signal, with a minimum background value of 50 arbitrary units established to prevent inflated ratios resulting from division by very low values. The final data were expressed as fluorescence intensity ratio (sample/background signal) in arbitrary units [A.U.]. All HuProt™ Human Proteome Microarray assays, including data acquisition, analysis, and validation, were overseen by experts from PEPperPRINT GmbH (Heidelberg, Germany) to ensure the reliability of the primary raw data.

To identify proteins potentially targeted by IgG, we applied dual selection criteria: (i) signal intensity exceeding three standard deviations above the mean (mean + 3 SD) of the healthy control group for each protein category and (ii) a fluorescence signal ratio greater than 2 when comparing HTLV-1-infected groups to healthy controls. These thresholds were selected as preliminary, heuristic cutoffs to balance sensitivity and specificity in this exploratory screening. The mean + 3 SD criterion helps reduce false-positive signals by identifying only those proteins with reactivity well above background variation observed in uninfected individuals. The >2 signal ratio further ensures that selected targets reflect substantial differential reactivity and are not merely influenced by minor fluctuations or low-abundance binding. To further evaluate differential IgG reactivity among proteins recognized by three or more samples, each HTLV-1–infected group was compared with the healthy control group. Group-wise signal intensities were analyzed using the Mann–Whitney U test, followed by Benjamini–Hochberg false discovery rate (FDR) correction, with statistical significance set at q < 0.05.

### 4.6. Protein–Protein Interaction Network (PPIN) Analysis

To evaluate the PPIN among targeted proteins, we analyzed the protein lists for each group using the STRING platform (V12.0, STRING Consortium, 2024). Structural homology and co-expression relationships were independently assessed for all identified proteins within each group. Additionally, functional enrichment analysis was performed using the same platform to identify statistically significant associations (FDR ≤ 0.05) with biological processes and molecular functions (Gene Ontology), reference publications (PubMed), local network clusters (STRING), Reactome pathways, WikiPathways, disease-gene associations (DISEASES), annotated keywords (UniProt), protein domains and features (InterPro), subcellular localization (COMPARTMENTS), and protein domains (SMART).

### 4.7. Statistical Analysis

All statistical analyses were performed using GraphPad Prism 5.0 (GraphPad Software, La Jolla, CA, USA). The in vitro experiments were conducted independently on five occasions, each using PBMCs from two different donors. Comparisons between groups were evaluated using Student’s *t*-test or the non-parametric Mann–Whitney U test, as appropriate. A *p*-value ≤ 0.05 was considered statistically significant.

## 5. Conclusions

In conclusion, this study suggests a potential association between the clinical manifestations of HTLV-1 infection and distinct cytokine production profiles in major peripheral T-cell subsets, as well as differential recognition of self-proteins by IgG idiotype repertoires. Our findings support the hypothesis that individuals with HAM/TSP or ATLL may develop unique sets of autoreactive IgG antibodies capable of targeting proteins expressed in immune cells and specific human tissues, potentially contributing to immune dysregulation and disease-specific pathogenesis.

## Data Availability

The full datasets used to produce the current study are available from the corresponding author upon reasonable request.

## Supplementary Materials

The following supporting information can be downloaded at: https://www.mdpi.com/article/10.3390/ijms262210858/s1.
