# Supplementary material for: IgG Idiotype Diversity Shapes Cytokine Profiles and Autoantibody Targets in HTLV-1 Clinical Outcomes
Source: Int J Mol Sci. 2025 Nov 8;26(22):10858. doi: 10.3390/ijms262210858 (PMC12652327; doi:10.3390/ijms262210858)
Supplement: Supplementary file 1 [file ijms-26-10858-s001.zip › Supplementary Table S1.pdf]

Supplementary Table S1: Number of potential recognized targets in the experimental groups of IgG donors, as determined by a ratio >2 compared to healthy control donors. The table also includes the cutoff value, calculated as the mean plus 3 standard deviations (S.D.).

Groups	Number of evaluated targets (expressed genes)	Number of possible recognized targets (ACs / HAM / ATLL)	N-exp HC Mean / S.D.	Threshold (mean of HC + 3 x S.D.)
**Immune system components**				
Antibodies	16	0 / 0 / 1	126 / 98	420
Antibodies receptors	18	0 / 0 / 1	203.9 / 153.5	664.4
Apoptosis	41	1 / 2 / 1	504.5 / 579.1	2241.8
Clusters of differentiation (CD)	84	0 / 0 / 1	273.8 / 308	1,197.8
Complement	34	0 / 0 / 0	487.9 / 1292	4363.9
Cytokines	120	0 / 0 / 0	207.6 / 318.3	1,162.5
Cytokines Receptors	84	0 / 1 / 0	200.3 / 337.9	1,214
**Immune system cells**				
B cell	270	0 / 1 / 6	294.9 / 574.8	2,019.3
Dendritic Cells	372	1 / 3 / 6	324.2 / 818.3	2,779.1
Monocyte	717	0 / 6 / 8	367.7 / 1,134	3,769.7
Natural Killer	444	0 / 5 / 2	367.1 / 1,301	4,270.1
T regulatory cell	239	0 / 2 / 3	335.8 / 1,505	4,850.8
TCD4 memory	116	0 / 3 / 3	251.7 / 368.9	1,358.4
TCD4 naive	150	0 / 5 / 4	354.6 / 617.8	2,208
TCD8 memory	122	0 / 3 / 3	265.6 / 382.6	1,413.4
TCD8 naive	145	0 / 3 / 3	269 / 389.6	1,437.8
Tγδ cell	174	0 / 3 / 3	284.8 / 512.5	1,822.3
**Tissues**				
Adipose Tissue	3	0 / 0 / 1	240 / 133.9	641.7
Adrenal Gland	19	0 / 0 / 0	281.6 / 321.3	1,245.5
Bone Marrow	87	0 / 1 / 3	259 / 377.9	1,392.7
Brain	374	1 / 8 / 11	262.4 / 497.3	1,754.3
Breast	16	0 / 0 / 0	423.6 / 739.8	2,643
Chroroid Plexus	29	0 / 0 / 0	152.6 / 117.1	503.9
Endometrium	3	0 / 0 / 0	120.6 / 126.3	499.5
Epididymis	73	0 / 1 / 0	226.9 / 525.4	1,803.1
Esophagus	17	0 / 0 / 0	403.9 / 582.9	2,152.6
Fallopian Tube	12	0 / 0 / 0	208.6 / 239	925.6
Gallbladder	3	0 / 0 / 0	96.37 / 72.18	312.91
Heart	26	0 / 0 / 0	429 / 883.6	3,079.8
Intestine	92	0 / 0 / 1	176.3 / 264.9	971
Kidney	52	1 / 3 / 2	180.4 / 160.5	661.9
Liver	238	1 / 1 / 2	208.4 / 404.9	1,423.1
Lung	16	0 / 1 / 1	165.8 / 93.38	445.94
Lymphoid Tissue	100	1 / 3 / 4	165.3 / 161.1	648.6
Ovary	4	0 / 0 / 0	256.3 / 158.2	730.9
Pancreas	59	0 / 0 / 0	197.7 / 359.3	1,275.6
Parathyroid Gland	21	0 / 0 / 0	325.8 / 781	2,668.8
Pituitary Gland	22	0 / 0 / 1	146.2 / 142.5	573.7
Placenta	53	0 / 0 / 2	280.7 / 474.4	1,703.9
Prostate	12	0 / 0 / 1	243.5 / 259.4	1,021.7
Retina	96	2 / 1 / 1	235 / 419.2	1,492.6
Salivary Gland	31	0 / 0 / 1	493.3 / 1281	4,336.3
Seminal Vesicle	4	0 / 1 / 1	82.68 / 42.69	210.75
Skeletal muscle	34	1 / 1 / 2	182.6 / 224.5	856.1
Skin	128	0 / 4 / 1	172.7 / 204	784.7
Stomach	27	0 / 0 / 0	458.7 / 1234	4,160.7
Testis	547	2 / 14 / 7	224.6 / 514.2	1,767.2
Thyroid Gland	11	0 / 0 / 0	98.84 / 61.63	283.7
Urinary Bladder	5	0 / 0 / 0	286.9 / 215.7	934
**Tissue-shared proteins**				
Several	465	3 / 6 / 9	263.1 / 500.8	1,765.5
